# Influence of Chewing Rate and Food Composition on *in Vivo* Aroma Release and Perception of Composite Foods

**DOI:** 10.1021/acs.jafc.3c09346

**Published:** 2024-03-13

**Authors:** Karina Gonzalez-Estanol, Michele Pedrotti, Mònica Fontova-Cerdà, Iuliia Khomenko, Franco Biasioli, Markus Stieger

**Affiliations:** † Research and Innovation Centre, Edmund Mach Foundation, 38098 San Michele All’Adige (TN), Italy; ‡ Food Quality and Design, Wageningen University, 6708 WG Wageningen, The Netherlands; § Department of Agri-Food and Environmental Sciences, Trento University, I-38123 Trento, Italy; ∥ Division of Human Nutrition and Health, Wageningen University, 6708 WE Wageningen, The Netherlands

**Keywords:** time-intensity (TI), aroma release, proton-transfer reaction-time-of-flight mass spectrometry (PTR-ToF-MS), composite foods, oral processing behavior

## Abstract

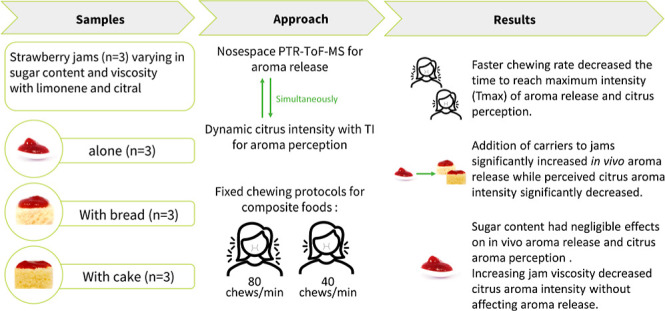

This study investigated the effects of chewing rate and food composition on *in vivo* aroma release and perception of composite foods. Bread or sponge cake paired with varying sugar content and viscosity strawberry jams, spiked with citral and limonene, were examined. In-nose release was characterized using Proton-Transfer-Reaction-Time-of-Flight-Mass-Spectrometry (PTR-ToF-MS). Simultaneously, Time-Intensity (TI) profiling assessed citrus aroma perception (*n* = 8, triplicate) while fast and slow chewing protocols were applied (fast: 1.33 chews/s; slow 0.66 chews/s; each for 25 s). Chewing rate did not significantly impact the area under the curve and maximum intensity of *in vivo* citral and limonene release and citrus aroma perception. Faster chewing rates significantly decreased the time to reach maximum intensity of aroma release (*p* < 0.05) and citrus aroma perception (*p* < 0.001). Faster chewing rates probably accelerated structural breakdown, inducing an earlier aroma release and perception without affecting aroma intensity. Adding carriers to jams significantly (*p* < 0.05) increased aroma release, while perceived citrus aroma intensity significantly (*p* < 0.05) decreased regardless of chewing rate. In conclusion, chewing rate affects the temporality of *in vivo* aroma release and perception without affecting its intensity, and carrier addition increases *in vivo* aroma release while diminishing aroma perception.

## 1 Introduction

The release of volatile organic compounds (VOCs) from the food matrix into the oral and nasal cavities during consumption is crucial for aroma perception. This complex phenomenon, influenced by various factors, depends on oral processing behavior. When consuming solid foods, the number of bolus fragments increases and their size decreases, leading to an increase in the total surface area of food bolus particles, which facilitates the release of taste and aroma compounds from the food matrix, potentially enhancing taste and aroma perception.^1,2^

Several studies explored the impact of oral processing behavior on *in vivo* aroma release. For instance, Tarrega et al. (2008) found positive correlations between the number of chews, chewing work, and chewing strength, with the maximum concentration of released aroma compounds in cheeses. Furthermore, the time to reach the maximum concentration was correlated with chewing time.^3^ Feron et al. (2014) emphasized the significance of masticatory behavior in cheeses, with chewing amplitude having an impact on aroma release after swallowing.^4^ Repoux et al. (2012) showed that firmer processed cheeses led to longer chewing durations and increased *in vivo* aroma release.^5^ More recently, Okawa et al. (2021) reported positive correlations between aroma nose space concentration, the number of chewing strokes, and salivary flow rate during mastication of gummy jellies.^6^ How et al. (2021) demonstrated that *in vivo*. aroma release from cooked white rice was influenced by particle breakdown pathways, where bolus with smaller particles resulted in a higher aroma release.^42^ While these studies consistently showed that variations in oral processing behaviors influenced *in vivo* aroma release, they did not quantify the impact of these differences on aroma perception. Therefore, it is not evident from these studies how differences in *in vivo* aroma release resulting from distinct oral processing behaviors translate into differences in aroma perception.

Luckett et al. (2016, 2017) showed that the number of chews and chewing rate modulate the dynamic flavor perception of potato chips.^7,8^ They observed that perceived maximum flavor intensity and area under the Time-Intensity (TI) curve were higher for medium and fast chewing rates than for slow chewing rates,^8^ suggesting that increased oral structural breakdown of potato chips increased flavor perception. Tian et al. (2023) evaluated the rate of flavor release in the mouth during consumption of dry-cured pork and showed that the interaction between pork and saliva caused changes in sensory perception.^9^ Doyennette et al. (2019) showed that “chewers” consumed ice creams with a shorter consumption time and perceived aromas earlier and longer compared to “melters”, who consumed ice creams slower,^10^ demonstrating the impact of oral behavior on aroma perception of ice creams. Devezeaux de Lavergne et al. (2015) showed that variations in individual eating behaviors, such as short- and long-duration chewing, resulted in different bolus properties of sausages, ultimately leading to differences in dynamic texture perception of the same sausage.^11^ While these studies demonstrated the impact of oral processing behavior on flavor perception, these studies did not quantify aroma release during consumption. It is hence unclear whether the observed differences in aroma perception are linked to changes in aroma release resulting from variations in oral behavior.

To summarize, the studies described above assessed the influence of oral processing behaviors on either *in vivo* aroma release or aroma perception. Only few studies integrated both methodologies simultaneously to assess the influence of oral processing behaviors on *in vivo* aroma release and perception. Déléris et al. (2011) demonstrated that chewing gelatin gels led to an earlier *in vivo* aroma release and perception compared to letting gelatin gels melt in the mouth.^12^ Leclercq and Blancher (2012) investigated the benefits of imposing a strict chewing and swallowing protocol on *in vivo* aroma release and perception of flavored gelled candies. They highlighted the effect of interindividual variability on aroma release.^13^ Recently, it was shown that extending the chewing period to longer time periods enhanced *in vivo* aroma release and optimized the consumer experience of grilled eel.^14^

While these studies have offered valuable insights into the impact of oral processing behaviors on *in vivo* aroma release and perception, there exists a research gap requiring a broader exploration involving more complex foods, such as composite foods. Commonly consumed foods often encompass various components that are consumed together, referred to as composite foods. For instance, bread or wafer (carrier foods) are frequently consumed alongside spreads or toppings. The compositional, mechanical, and sensory attributes of carrier foods differ considerably from those of spreads or toppings.^15^ The exploration of composite foods is gaining significance not only due to their increased sensory complexity but also because they provide sensory profiles that closely align with their natural consumption contexts. To the best of our knowledge, only three studies delved into the *in vivo* aroma release and perception of composite foods.^16,17^ However, these studies did not assess the influence of oral processing behavior on *in vivo* aroma release and perception of composite foods.

Enhancing our understanding of how oral processing behaviors influence aroma release and perception of composite foods has the potential to increase the practical applicability of acquired sensory and aroma release profiles. This study aimed to investigate the effects of (a) chewing rate (fast vs slow) and (b) carrier addition (bread, sponge cake) on *in vivo* aroma release and perception of strawberry jams varying in composition. Nose space analysis with Proton-Transfer- Reaction-Time-of-Flight-Mass-spectrometry (PTR-ToF-MS) coupled with dynamic sensory analysis through TI profiling was used. It is hypothesized that (a) aroma release and perception are affected by chewing rate, with faster chewing rate facilitating the breakdown of the food bolus into more and smaller fragments, thereby promoting aroma release from the food matrix into the nasal cavity and resulting in higher aroma intensity perception compared to a slower chewing rate. Additionally, (b) the addition of solid carriers to strawberry jams is expected to increase aroma release while diminishing aroma perception.

## 2 Materials and Methods

### 2.1 Samples

Three strawberry jams with variations in sugar content and viscosity were prepared (Menz & Gasser, Novaledo Italy). These jams encompassed high sugar/medium viscosity (HS/MV), high sugar/high viscosity (HS/HV), and low sugar/low viscosity (LS/LV) formulations, with specific details provided in Table 1. Reformulation of the strawberry jams adhered to pragmatic limits for product reformulation, ensuring their close resemblance to commercially available products. All strawberry jams were spiked with 0.4% (w/w) citral (Sigma-Aldrich, USA) and 0.4% (w/w) limonene (Sigma-Aldrich, USA). The selection of citral and limonene as markers was based on a previous study investigating *in vivo* aroma release and citrus aroma perception.^16^ Despite both compounds sharing a citrus aroma, they differ in physicochemical properties with citral showing a molecular weight (Mw) of 152 g/mol and a log*P* value of 2.76, while limonene has a Mw of 136 g/mol and a log*P* of 4.20. The concentrations of citral and limonene were determined through pilot trials to ensure that participants clearly recognized the citrus aroma in the strawberry jams and to ensure that a robust PTR-ToF-MS signal was obtained. In the pilot study, a range of concentrations of citral and limonene were added to strawberry jams. Participants were asked to assess the perceived intensity of the citrus aroma. Citral and limonene concentrations above 0.4% (w/w) resulted in high peaks in PTR-ToF-MS but tended to provoke an artificial flavor. Conversely, concentrations below 0.2% (w/w) gave a weak citrus aroma intensity and were deemed unsuitable. Therefore, concentrations of 0.4% (w/w) for citral and limonene were chosen as the pilot study demonstrated that at this concentration, a citrus aroma was clearly perceived by participants while ensuring a good signal in PTR-ToF-MS. Composite foods were formed by combining the three strawberry jams with two carriers (bread and sponge cake). The selection of these carriers aimed to replicate the usual consumption context of strawberry jams and because of their differences in mechanical properties (Table 1).

**Table 1:** Sugar Content and Rheological Characteristics of Strawberry Jams together with Mechanical Properties of the Carriers

	strawberry jams			carriers	
	high sugar/medium viscosity (HS/MV)	low sugar/low viscosity (LS/LV)	high sugar/high viscosity (HS/HV)	bread (B)	sponge cake (SC)
sugarcontent (g/100 g)	52	39	52		
Brix	60	45	60		
viscosity at a shear rate of 1.5 s^–1^ (Pa·s)	52	18	98		
hardness (N)				509 ± 37	10 ± 1

**Figure 1:**
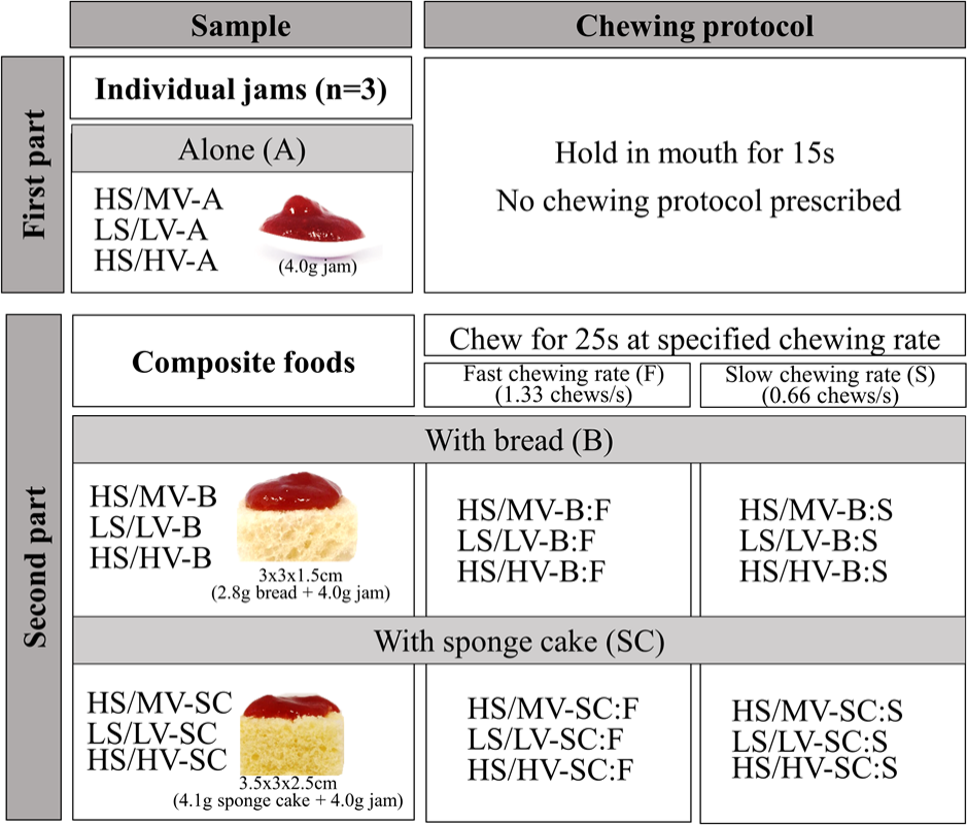
Experimental design outlining all samples used. In part one, jams varying in sugar content (HS: high sugar; LS: low sugar) and viscosity (LV: low viscosity; MV: medium viscosity; and HV: high viscosity) were evaluated on their own (alone: A) without a prescribed chewing protocol. In part two, composite foods (B, bread; SC, sponge cake) were evaluated using fast and slow chewing rates (F, fast chewing rate of 1.33 chews/s for 25 s; S, slow chewing rate of 0.66 chews/s for 25 s).

Commercially available white bread (Bruschelle mini, Morato, Italy) was used. Bread was cut into pieces of 3 × 3 × 1.5 cm without crust (2.8 ± 0.6 g), and strawberry jam (4.0 ± 0.3 g) was spread on top. Prepacked sponge cakes (Soremartec, Alba, Italy) were used. Sponge cakes were cut into pieces of 3.5 × 3.0 × 2.5 cm without crust (4.1 ± 0.6 g), and strawberry jam (4.0 ± 0.5 g) was spread on top. The mass ratios of bread/jam and sponge cake/jam were determined in a preliminary study (data not shown) in which participants were asked to spread strawberry jams on top of breads or sponge cakes as they normally do, maintaining a bite size for comfortable consumption. The observed mass difference is attributed to the distinct densities of the two carrier products. All samples were prepared shortly before serving (<30 min). A description of sample codes and pictures is provided in Figure 1.

### 2.2 Participants

Nine Caucasian women were recruited from the Edmund Mach Foundation (San Michele all’Adige, Trentino, Italy). The eligibility criteria included the absence of allergies or intolerances to wheat/gluten, dairy, nuts, soybeans, eggs, nonpregnant and nonlactating status, no history of oral perception disorders or olfactory impairments, and not being on a calorie-restricted diet (self-reported). Before starting the study, participants provided written informed consent and received financial compensation for their time in the form of a gift coupon upon completion of the study. The study adhered to the ethical guidelines outlined in the Declaration of Helsinki (2013). Among the initially recruited participants, eight participants (age 27 ± 5 years, all females, and BMI 20.9 ± 1.9 kg/m^2^) successfully concluded the study.

### 2.3 Assessment of Bite Size, Consumption Time, and Chewing Protocol

Bite size was based on previous studies.^18^ Additionally, input from a focus group comprising 10 female participants not taking part in the primary study was incorporated to validate the selected bite sizes.

This focus group also defined both, the total consumption time and the chewing rate protocols for all samples. Following the procedure previously described,^18−20^ individual video recordings were conducted to characterize oral processing behaviors. Participants were presented with fixed bite sizes of jam alone (4.0 g), jam–bread combinations (6.8 g), and jam–sponge cake combinations (8.2 g). Participants were instructed to chew in a normal manner and to indicate when they swallowed. Consumption time (s), defined as the average time from introducing the sample in the mouth until swallowing, and number of chews per bite (−), calculated from vertical jaw displacement, were extracted from video recordings. Chewing rate (chews/s) was subsequently calculated. The derived averages of these parameters served to establish the chewing protocols adopted in the study. Based on this preliminary study, the total consumption time was set to 15 s for jams consumed alone and 25 s for all composite foods. The chewing rates of the composite foods were set to 1.33 and 0.66 chews/s for 25 s, corresponding to fast and slow chewing, respectively.

### 2.4 Familiarization Sessions

Participants took part in two 1 h familiarization sessions. The initial session focused on the recognition and assessment of strawberry jams with varying citrus aroma intensities. This involved using references: one jam without added citrus aroma (not citrus at all) and another jam spiked with 1.25% (w/w) limonene and citral (extremely citrus). Subsequently, participants were introduced to the TI methodology (Section 2.5) and practiced with strawberry jam spiked with 0.2% w/w citral and 0.2% w/w limonene, allowing them to familiarize themselves with the intensity scale and the TI procedure. The session concluded with participants practicing the different chewing protocols using a metronome to adhere to the specified chewing frequencies. In the subsequent familiarization session, participants evaluated the samples following the procedures outlined in the actual experiment (Section 2.6). This session aimed to enhance participants’ comfort with the overall setup, the prescribed chewing protocol, and the TI task.

### 2.5 TI Profiling

The evaluation of dynamic citrus aroma intensity in strawberry jams was done using the TI methodology (*n* = 8; triplicate). In this context, citrus aroma was defined as “the combination of aromas associated with citrus fruits such as lemon, lime, orange, tangerine, and grapefruit”. Participants were given specific instructions to insert the sample into their mouths, to initiate the evaluation by clicking on the “start” button on a screen placed in front of them, and to promptly start tracking citrus aroma intensity. Throughout the evaluation period, participants were instructed to move a cursor along a 100 mm unstructured horizontal line scale, anchored from not at all to extreme citrus aroma intensity (EyeQuestion software, version 5). If participants perceived any differences during the evaluation, they were instructed to adjust the cursor accordingly.

Clear instructions regarding the chewing rate (1.33 or 0.66 chews/s) and the moment of swallowing (15 s for jam alone, 25 s for composite foods) were provided to the participants during the TI profiling using a metronome and a visual prompt displayed on a computer screen. TI data was recorded every second, concluding 135 s after participants clicked the “start” button.

### 2.6 *In Vivo* Nose Space Analysis and Dynamic Sensory Evaluation with TI

The experimental protocol for assessing *in vivo* nose space release of citral and limonene from strawberry jams was adapted from previous PTR-ToF-MS nose space studies.^16,21,22^ Using a commercial PTR-ToF-MS 8000 instrument (Ionicon Analytik GmbH, Innsbruck, Austria), the ionization conditions were set to 628 V drift voltage, 110 °C drift temperature, and 2.80 mbar drift pressure, resulting in E/N = 130 Td. Data acquisition was at a rate of one spectrum per second with an inlet flow of 500 sccm. The NASE sampling system (Ionicon Analytik GmbH, Innsbruck, Austria) was employed to sample from both nostrils. This system was heated to 110 °C and directly connected to a polyetheretherketone inlet maintained at the same temperature. All of the evaluations were conducted individually in a laboratory setting with filtered air. For each sample, participants were instructed to insert the tubes into their nostrils and breathe normally through their nose with their mouth closed. After 60 s of sampling their breath, participants were prompted to place the entire sample in their mouth, click the “start” button on the screen, and start the TI evaluation with their mouth closed. Simultaneous acquisition of TI and nose space data occurred for 135 s (*n* = 8; triplicate).

### 2.7 Experimental Procedure

TI and nose space analyses for all samples were performed during five sessions, each lasting 60 min. These sessions were divided into two parts (Figure 1). In the first part, composed of one session, participants evaluated the three jams without carries in triplicate. The samples were organized into blocks of three jam formulations (HS/MV-A, LS/LV-A, and HS/HV-A), and the order of jam formulations within each block was randomized for each participant. Participants evaluated the samples monadically, keeping the jam in their mouths for 15 s before swallowing. No specific chewing protocol was imposed.

The second part comprised four sessions, each lasting 60 min, during which participants evaluated the composite foods following the two chewing protocols. In each session, participants assessed a single carrier-chewing rate combination (B:F, B:S, SC:F, and SC:S) with the three jam formulations (HS/MV, LS/LV, and HS/HV) in a randomized order, in triplicate. The order of these four sessions was counterbalanced within participants. Participants were instructed to chew the composite foods for 25 s before swallowing, adhering to the different chewing rates with the help of a metronome and visual prompts on a computer screen.

All samples were served at room temperature (21 ± 1 °C) in standardized bite sizes (Figure 1) and were coded with random three-digit numbers. Between samples, participants took 3 min breaks, during which they could have a sip of water and eat a cracker to cleanse their palate. Participants were instructed to abstain from eating, drinking anything except for water, or using any persistent flavored product for at least 1 h before the start of the session. Additionally, there was a washout period of at least 2 days between the first and second part of the study.

### 2.8 Data Analysis

#### 2.8.1 TI Data Analysis

To evaluate the effect of chewing rate, citrus aroma intensity values of composite foods were averaged across participants for each second of the evaluation period, resulting in average TI curves for each sample and chewing rate. These average TI curves serve the purpose of visualizing the effects of the chewing rate on the temporal perception of citrus aroma. Additionally, TI curves were built for each participant and replicate and TI key parameters such as area under the curve (AUC), maximum aroma intensity (*I*_max_), time to reach maximum aroma intensity (*T*_max_), rising slope of the TI curve (*R*_i_) defined as rate of intensity increase (linear fit of TI data from 0 s to *T*_max_), and declining slope of the TI curve (*R*_f_) defined as the rate of intensity decrease (linear fit of TI data from *T*_max_ to time point when the baseline is reached) were extracted from these individual TI curves. Extracted parameters were subsequently subjected to statistical data analysis. To analyze the impact of chewing rate on AUC, *I*_max_ and *T*_max_, and *R*_i_ and *R*_f_, individual linear mixed models (LMM) were employed for each extracted parameter. The LMMs were applied to composite foods, considering chewing rate (fast/slow), carrier (bread, sponge cake), jam formulation (HS/MV, LS/LV, and HS/HV), and their interaction as fixed effects. Single observations per participant were treated as random effects.

To evaluate the impact of carrier addition and jam formulation on citrus aroma intensity, values for both individual and composite foods were averaged across participants and chewing rates, resulting in TI curves. Consistent with the previous approach, these average curves were generated for visualization of the effects of the carrier and formulation on citrus aroma perception. Additioanlly, curves were built for each participant and replicate and AUC, *I*_max_, *T*_max_, *R*_i_, and *R*_f_, were extracted from these individual TI curves. Extracted parameters were used for subsequent statistical analysis. Individual LMMs were performed for each extracted parameter. LMMs were applied to individual and composite foods. Carrier (non, bread, and sponge cake), jam formulation (HS/MV, LS/LV, and HS/HV), and their interaction were considered as fixed effects. Single observations per participant were treated as random effects.

For all LMMs, post hoc tests were performed with Tukey’s honest significant difference (HSD) test at a 95% confidence level. The curves were smoothed using the “smoothing.spline” function in the TempR package. Plots were created using “ggplot”, and standard deviation (SD) was added as a “geom_ribbon” to the plots (R software; version 3.1.1).

#### 2.8.2 *In Vivo* Nose Space Data Analysis

PTR-ToF-MS data were processed with in-house software (Sensory Quality Unit, Edmund Mach Foundation) as described elsewhere.^23^ Peak identification was performed using an in-house library developed by the authors. Mass peaks corresponding to the isotope of limonene (*m*/*z* 138.13) and citral (*m*/*z* 153.13) were extracted, and their concentrations were calculated. The isotope was chosen due to the abundant concentration of mass peak corresponding to limonene that led to detector saturation. Similar to the data analysis of the TI curves, to visualize the effect of chewing rate on *in vivo* nose space release, values of limonene and citral release of composite foods were averaged over participants for every second of the evaluation period, and aroma release curves were obtained for each sample and chewing rate. To determine carrier addition and formulation effects, limonene and citral release values were aggregated across participants and chewing rates. To assess the impact of chewing rate, carrier, and formulation on AUC, *I*_max_, *T*_max_, *R*_i_, and *R*_f_, a similar data analysis as detailed in Section 2.8.1 was followed.

## 3 Results

### 3.1 Influence of Chewing Rate on *In Vivo* Aroma Release and Perception

**Figure 2:**
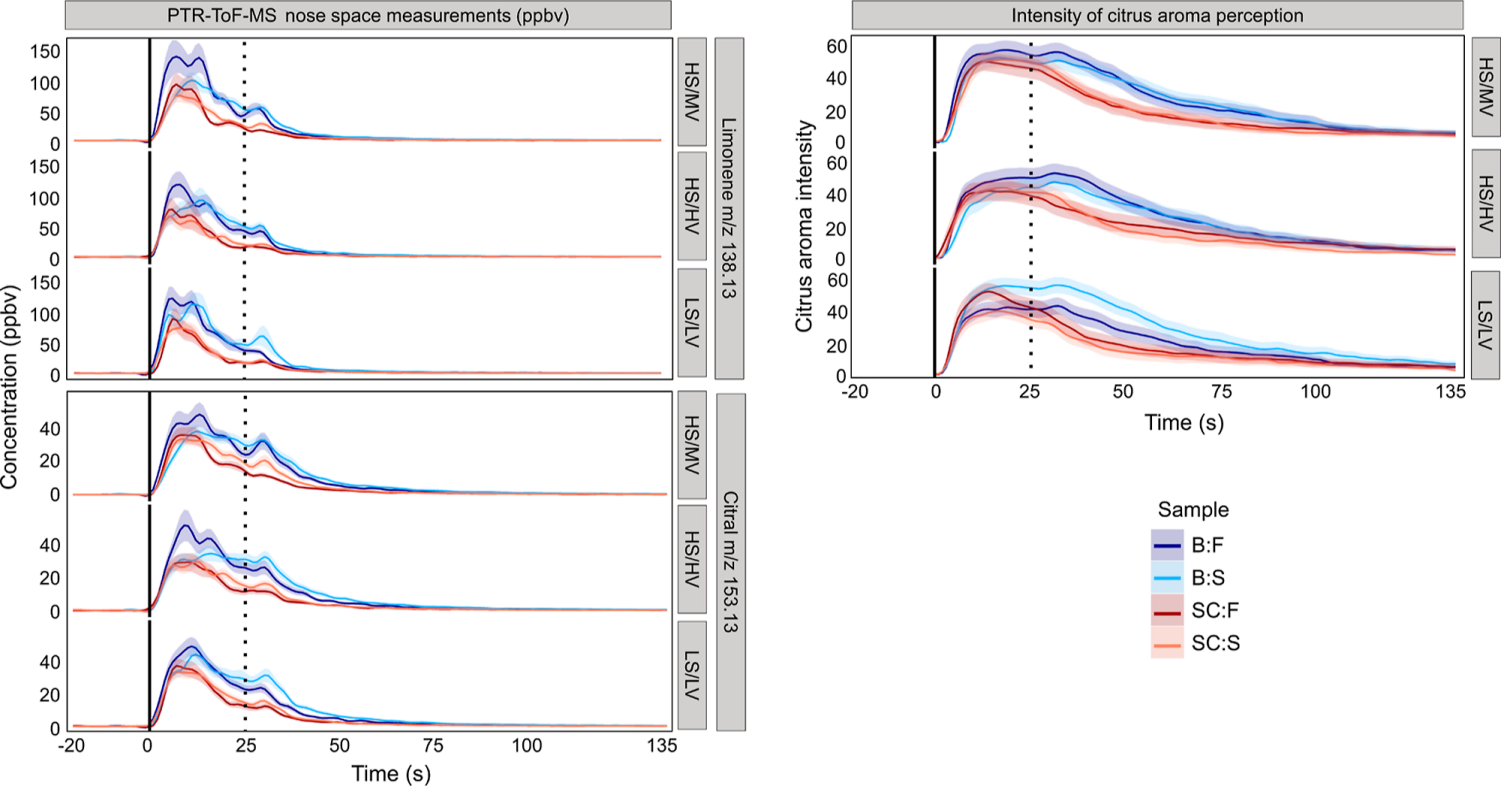
Aggregated data (*n* = 8, triplicate) for PTR-ToF-MS nose space measurements of limonene and citral concentration (ppbV) (left) and intensity of citrus aroma perception (right) for fast (darker shades) and slow (lighter shades) chewing rates [fast (F): chewing rate of 1.33 chews/s for 25 s; slow (S): chewing rate of 0.66 chews/s for 25 s] for composite foods (bread (B) with jams [shades of blue] and sponge cake (SC) with jams [shades of red]) for each jam formulation (HS/MV: high sugar/medium viscosity; HS/HV: high sugar/high viscosity; and LS/LV: low sugar/low viscosity). Black solid lines represent moments when samples were put in mouth, and dotted lines indicate the moment of swallowing.

Figure 2 shows the effect of chewing rate [fast (F): chewing rate of 1.33 chews/s for 25 s; slow (S): chewing rate of 0.66 chews/s for 25 s] on *in vivo* aroma release of limonene (*m*/*z* 138.13), citral (*m*/*z* 153.13), and the corresponding perception of citrus aroma intensity of different jam formulations (HS/MV, LS/LV, and HS/HV) consumed with carriers [bread (B) and sponge cake (SC)].

In terms of citrus aroma perception, no clear effect of the chewing rate on citrus aroma perception was observed as all curves exhibited a similar shape and overlapped. However, jams with bread (shades of blue) were generally perceived as more intensive in citrus aroma compared to jams with sponge cake (shades of red). Interestingly, the LS/LV jam with bread evaluated with a slow chewing rate, seemed to yield the highest aroma intensity, persisting until the end of the evaluation. Moreover, postswallowing, jams with sponge cakes showed a steeper decrease compared to jams with breads, where citrus perception lingered until the end of the evaluation.

Table 2 summarizes the parameters (AUC, *I*_max_, *T*_max_, *R*_i_, and *R*_f_) extracted from the individual *in vivo* aroma release curves for limonene and citral and the individual TI curves showing the effects of chewing rate, reformulation, and carrier addition on aroma release and perception. Additionally, Supporting Information Table S1 reports the corresponding results of the LMM considering the chewing rate, carrier, reformulation, and their interactions as fixed effects.

**Table 2:** Statistical Summary (Mean ± SD) of Composite Food for Area under the Curve (AUC), Maximum Aroma Intensity (*I*_max_), Time to Reach Maximum Aroma Intensity (*T*_max_), Rising Slope (*R*_i_), and Decline Slope (*R*_f_) Extracted from Individual Aroma Release Curves of Limonene (*m*/*z* 138.134) and Citral (*m*/*z* 153.128) and from Individual Citrus Aroma Perception Curves (TI Profiling)^a^

		AUC			*I*_max_			*T*_max_			increase rate			decrease rate		
		limonene	citral	citrus aroma perception	limonene	citral	citrus aroma perception	limonene	citral	citrus aroma perception	limonene	citral	citrus aroma perception	limonene	citral	citrus aroma perception
composite foods	HS/MV-B:F	2879.9 ± 349d	1382.3 ± 148c	3361 ± 2419 cd	206.2 ± 31.3c	60.4 ± 8.3bc	60.7 ± 28bc	11.9 ± 1.4bc	14.3 ± 1.6bcd	20.8 ± 11.3abc	24.49 ± 3.48ab	5.16 ± 0.81ab	4.97 ± 0.83ab	–0.55 ± 0.05a	–0.25 ± 0.02a	–0.49 ± 0.04a
	LS/LV-B:F	2396.4 ± 233.9bcd	1213.2 ± 96.8bc	3124 ± 2189bcd	178.6 ± 19.1bc	55.9 ± 5.3abc	56.7 ± 28.1abc	8.3 ± 0.9ab	10.9 ± 1.1ab	24.8 ± 10.5bc	25.88 ± 3.48b	6.07 ± 0.81ab	4.19 ± 0.83ab	–0.49 ± 0.05a	–0.23 ± 0.02abc	–0.36 ± 0.04abc
	HS/HV-B:F	2333.3 ± 287.6bcd	1307.6 ± 156.4c	2498 ± 1908abcd	160.2 ± 22.3abc	61.6 ± 9.2c	50.8 ± 23.5abc	9.7 ± 1.2ab	12.1 ± 1.3abc	18.9 ± 11.6abc	23.52 ± 3.48ab	6.85 ± 0.81b	3.25 ± 0.83ab	–0.47 ± 0.05ab	–0.25 ± 0.02a	–0.44 ± 0.04abc
	HS/MV-B:S	2470 ± 154.7d	1343.3 ± 106.3c	2382 ± 2227abc	153.6 ± 13abc	54.5 ± 3.7abc	51.9 ± 27abc	16 ± 1.5c	17.9 ± 1.7de	13.6 ± 6.8a	11.26 ± 3.32a	3.61 ± 0.78a	3.89 ± 0.8ab	–0.44 ± 0.05abc	–0.25 ± 0.02a	–0.43 ± 0.04abc
	LS/LV-B:S	2593.3 ± 279.3d	1326.2 ± 132.2c	2328 ± 2462abc	173.5 ± 22bc	55.2 ± 6abc	46.7 ± 29.3abc	11.9 ± 1.3bc	15.7 ± 1.6cde	16 ± 10.4ab	18.21 ± 3.32ab	4.42 ± 0.78ab	2.92 ± 0.8ab	–0.52 ± 0.05a	–0.24 ± 0.02a	–0.47 ± 0.04ab
	HS/HV-B:S	2342.5 ± 253.3 cd	1308 ± 131.2c	2239 ± 2056abc	148.1 ± 21.6abc	52.5 ± 5.4abc	53.9 ± 23.7abc	16 ± 1.3c	19.3 ± 1.4e	11.9 ± 7.4a	12.29 ± 3.32a	3.84 ± 0.78a	2.61 ± 0.8a	–0.42 ± 0.05abcd	–0.23 ± 0.02ab	–0.41 ± 0.04abc
	HS/MV-SC:F	1482.1 ± 200.4ab	815.6 ± 72ab	3248 ± 2529 cd	131.3 ± 23.9abc	47 ± 5.2abc	55.1 ± 25.2abc	9.1 ± 0.7ab	10.6 ± 0.9ab	23.3 ± 11.8bc	17.35 ± 3.48ab	6.11 ± 0.81ab	4.22 ± 0.83ab	–0.28 ± 0.05bcd	–0.15 ± 0.02 cd	–0.34 ± 0.04abc
	LS/LV-SC:F	1337.1 ± 180.3a	775.3 ± 77.1a	2829 ± 2427abcd	111.3 ± 17.7ab	45.8 ± 4.6abc	50.5 ± 28.5abc	7.2 ± 0.5a	9.3 ± 0.9a	26.5 ± 11.5c	17.8 ± 3.48ab	6.43 ± 0.81ab	5.81 ± 0.83b	–0.27 ± 0.05 cd	–0.15 ± 0.02 cd	–0.31 ± 0.04c
	HS/HV-SC:F	1336.4 ± 277.2a	733.3 ± 104.8a	3481 ± 2412d	108 ± 25.7ab	40.3 ± 5.6ab	61.5 ± 24.6c	9.1 ± 1.2ab	11.3 ± 1.2ab	25.5 ± 14.1bc	13.83 ± 3.48ab	4.65 ± 0.81ab	4.9 ± 0.85ab	–0.24 ± 0.05d	–0.13 ± 0.02d	–0.31 ± 0.04c
	HS/MV-SC:S	1529.4 ± 116.5abc	949.2 ± 73.8abc	2362 ± 2054abc	111.1 ± 12.6ab	47.5 ± 3.4abc	55.5 ± 27.6abc	12 ± 1.4bc	12.7 ± 1.4abc	17 ± 8.8ab	12.63 ± 3.32ab	4.67 ± 0.78ab	3.73 ± 0.8ab	–0.29 ± 0.05bcd	–0.18 ± 0.02abcd	–0.37 ± 0.04abc
	LS/LV-SC:S	1413 ± 131.6ab	830.6 ± 57.3ab	2058 ± 1899ab	113 ± 15ab	44.9 ± 3.4abc	46.9 ± 30.1ab	10.3 ± 1.2ab	12.3 ± 1.3abc	18.1 ± 10.9abc	13.96 ± 3.32ab	4.42 ± 0.78ab	3.84 ± 0.8ab	–0.27 ± 0.05 cd	–0.15 ± 0.02 cd	–0.29 ± 0.04c
	HS/HV-SC:S	1322.8 ± 164.2a	805.5 ± 81ab	1815 ± 2013a	94.5 ± 16.2a	38 ± 4.1a	44.8 ± 24.5a	9.6 ± 1.1ab	11.5 ± 1.3abc	19 ± 21abc	14.53 ± 3.32ab	4.68 ± 0.78ab	3.55 ± 0.8ab	–0.27 ± 0.05 cd	–0.16 ± 0.02bcd	–0.33 ± 0.04bc

a The data describes the impact of chewing rate (F: fast chewing rate of 1.33 chews/s for 25 s; S: slow chewing rate of 0.66 chews/s for 25 s). Lowercase letters within each column indicate statistically significant differences between means across samples, as discerned by Tukey’s HSD test for pairwise comparisons. The linear mixed models considered chewing rate (F:fast and S:slow), jam formulation (HS/MV: high sugar/medium viscosity; HS/HV: high sugar/high viscosity; and LS/LV: low sugar/low viscosity), carrier (B: bread and SC: sponge cake), and their interactions as fixed effects, with participants as a random effect.

The interaction effects between carrier, jam formulation, and chewing rate were significant for AUC (*F*_(2,267)_ = 3.23, *p* < 0.05), *I*_max_ (*F*_(2,268)_ = 6.22, *p* < 0.01), and *R*_f_ (*F*_(2,268)_ = 3.21, *p* < 0.05) of citrus aroma perception (Supporting Information Table S1). Notably, the AUC values of jams with breads were larger than the AUCs of jams with sponge cakes. Specifically, in the case of jams with breads, the highest AUC was found for LS/LV with a slow chewing rate, followed by HS/MV with a fast chewing rate. In contrast, for jams with sponge cakes, the highest AUC was attained by HS/MV with a fast chewing rate, followed by HS/MV with a slow chewing rate. The lowest AUC for jams with breads was observed for LS/LV during fast chewing, and in contrast, LS/LV with a slow chewing rate showed the lowest AUC for jams with sponge cakes. No clear trend was evident across samples for *I*_max_ of citrus aroma perception. LS/LV on bread with a slow chewing rate showed the highest intensity, followed by HS/MV on bread with a fast chewing rate. Conversely, the lowest *I*_max_ was observed in LS/LV on sponge cake with a slow chewing rate. Similarly, no clear trend was observed for *R*_f_, which decreased faster for jams with breads consumed with a slow chewing rate for HS/MV and HS/HV formulations and slower for jams with sponge cake for LS/LS consumed with a slow chewing rate and HS/HV consumed with fast chewing rate. The interaction between carrier and chewing rate was significant for *R*_i_ for limonene release (*F*_(1,269)_ = 5.96, *p* < 0.05). Jams with breads consumed with a fast chewing rate exhibited the highest rate of increase, in contrast to their slow chewing counterparts, which demonstrated the lowest rate of increase. None of the other interaction effects were significant (Supporting Information Table S1).

Chewing rate had a significant effect on *T*_max_. A faster chewing rate significantly reduced the *T*_max_ of limonene and citral release (limonene: *F*_(1,275)_ = 41.96, *p* < 0.001; citral: *F*_(1,273)_ = 43.45, *p* < 0.001). On average, there was a 27% reduction for limonene and a 23% reduction for citral of *T*_max_. Similarly, the faster chewing rate significantly decreased the *T*_max_ of the perception of citrus aroma (*F*_(1,276)_ = 10.8, *p* < 0.01) by 4 s (18% decrease of *T*_max_). There were no significant main effects of chewing rate of composite foods on AUC and *I*_max_ for the *in vivo* release of citral and limonene and the citrus aroma perception (Table 2). Lastly, chewing rate significantly impacted *R*_i_ for citral release (*F*_(1,273)_ = 18.74, *p* < 0.001) and citrus aroma perception (*F*_(1,274)_ = 8.24, *p* < 0.001) with fast chewing rate showing a faster increase compared to slow chewing rate.

### 3.2 Influence of Carrier Addition and Jam Formulation on *In Vivo* Aroma Release and Perception

**Figure 3:**
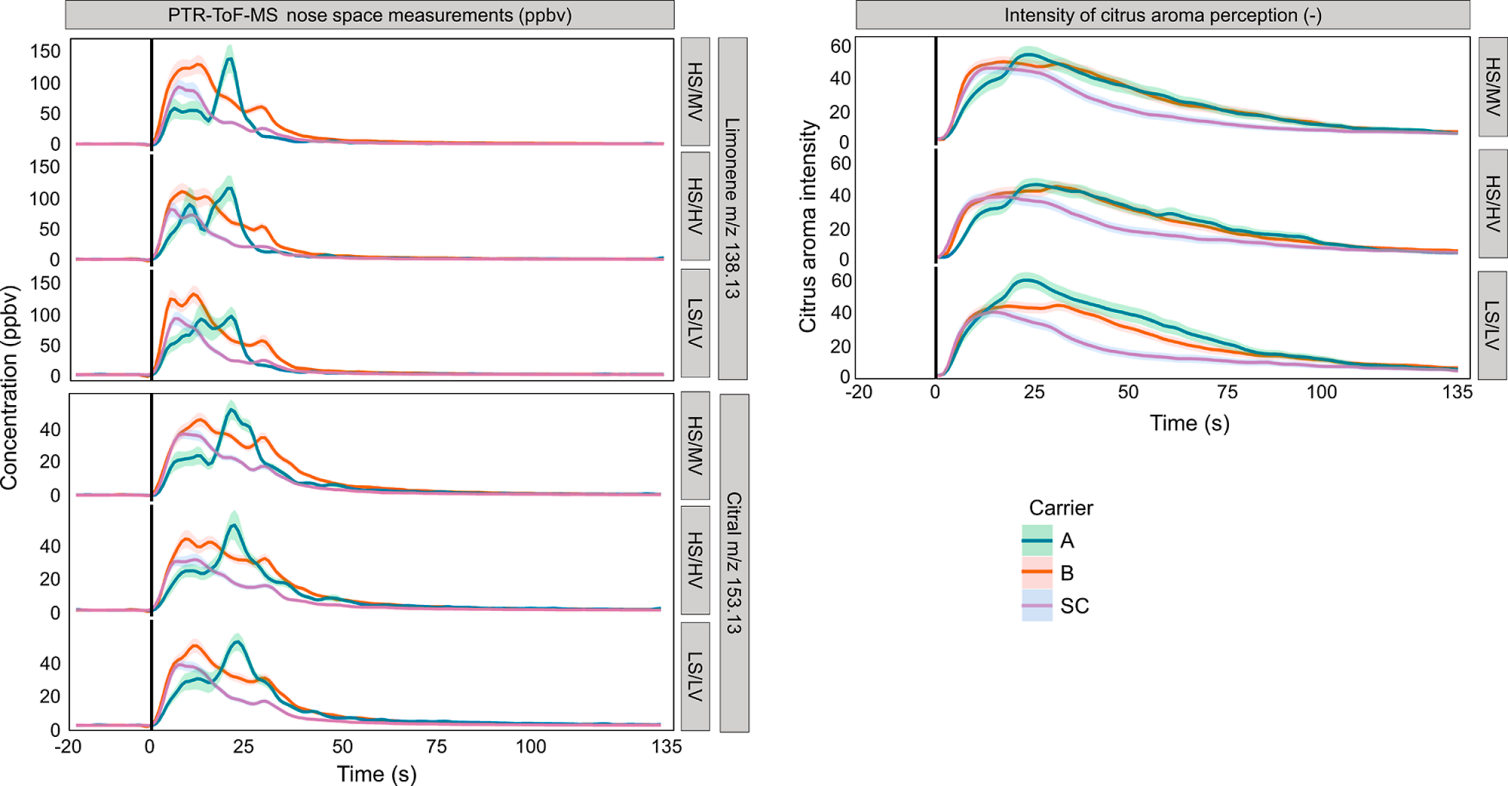
Aggregated data (*n* = 8, triplicate) for PTR-ToF-MS nose space measurements of limonene and citral concentration (ppbV) (left) and intensity of citrus aroma perception (right) for jams alone (A; green), bread with jams (B; red), and sponge cake with jams (SC; purple) for each jam formulation (HS/MV: high sugar/medium viscosity; HS/HV: high sugar/high viscosity; and LS/LV: low sugar/low viscosity). Black solid lines represent moments when samples were put in the mouth.

Figure 3 shows the effect of carrier addition (jams alone, jams with breads, and jams with sponge cake) on the *in vivo* aroma release of limonene and citral and the corresponding perception of citrus aroma intensity of different jam formulations (HS/MV, LS/LV, and HS/HV).

Carrier addition resulted in an initial increase in the release of limonene and citral during mastication (Figure 3). When jams were evaluated alone, citral and limonene release peaked after swallowing, corresponding to the swallow breath.^24^ In contrast, when jams were evaluated in combination with the carrier foods, a continuous decrease in release was observed after swallowing until the end of the evaluation. The TI curves revealed distinct trends in the citrus aroma intensity during consumption. In the initial periods of consumption, the intensity of citrus aroma exhibited a steeper increase for composite foods compared to that of jams evaluated alone. However, after the moment of swallowing, the intensity of composite foods began to decline, with sponge cake showing a more rapid decrease than that of bread. The citrus aroma intensity perception of jams with breads seemed to plateau until the midpoint of the evaluation and then gradually decreased towards the end. In summary, jams with breads or sponge cakes consistently exhibited a higher level of *in vivo* citral and limonene release compared to jams evaluated alone. The maximum perception of citrus aroma intensity of the composite foods was lower than that of jams evaluated alone during mastication. This pattern was consistently observed for all jam formulations (Figure 3).

Table 3 summarizes the parameters (AUC, *I*_max_, *T*_max,_*R*_i_, and *R*_f_) extracted from the *in vivo* aroma release curves for limonene and citral and from the individual TI curves showing the effects of carrier addition and jam formulation on individual and composite foods. Supporting Information Table S2 reports the corresponding results of the LMM considering carrier, jam formulation, and their interaction as fixed effects.

**Table 3:** Statistical Summary (Mean ± SD) of Individual Jams (A: Alone; HS/MV: High Sugar/Medium Viscosity; HS/HV: High Sugar/High Viscosity; and LS/LV: Low Sugar/Low Viscosity) and Composite Foods (B: Bread and SC: Sponge Cake) for Area under the Curve (AUC), Maximum Aroma Intensity (*I*_max_), Time to Reach Maximum Aroma Intensity (*T*_max_), Rising Slope (*R*_i_), and Decline Slope (*R*_f_) Extracted from Individual Aroma Release Curves of Limonene (*m*/*z* 138.134) and Citral (*m*/*z* 153.128), and from Individual Citrus Aroma Perception Curves (TI Profiling)^a^

		AUC			*I*_max_			*T*_max_			increase rate			decrease rate		
		limonene	citral	citrus aroma perception	limonene	citral	citrus aroma perception	limonene	citral	citrus aroma perception	limonene	citral	citrus aroma perception	limonene	citral	citrus aroma perception
individual jams	HS/MV-A	1629.2 ± 183.9ab	1032 ± 81.7abc	3146 ± 2113.7 cd	179.3 ± 19.7a	70.6 ± 5.2c	59.8 ± 28.8abc	17.1 ± 1.3de	19.9 ± 1.2 cd	21.6 ± 6.5abc	12.92 ± 3.32ab	3.84 ± 0.76ab	2.89 ± 0.73a	–0.31 ± 0.05bc	–0.201 ± 0.02abc	–0.47 ± 0.05ab
	LS/LV-A	1666.7 ± 208.9ab	1135.6 ± 117.5bc	3570.4 ± 1975.9d	173.2 ± 21.6ab	65.2 ± 5.3c	67.6 ± 27.3c	17.1 ± 1.3de	20.1 ± 1.1 cd	25.2 ± 11.6c	13.9 ± 3.32ab	2.99 ± 0.76ab	3.11 ± 0.73a	–0.31 ± 0.05bc	–0.214 ± 0.02ab	–0.55 ± 0.05a
	HS/HV-A	1723.6 ± 249.7abc	1078.1 ± 121.9abc	2919.7 ± 1629.5abcd	163 ± 25.2abc	66.2 ± 8.3c	55.9 ± 24.6abc	18 ± 1.5e	20.9 ± 1.2d	24.4 ± 8.2bc	10.27 ± 3.32a	2.51 ± 0.76a	2.52 ± 0.73a	–0.33 ± 0.05bc	–0.197 ± 0.02abc	–0.46 ± 0.05ab
composite foods	HS/MV-B	2662.9 ± 183.8d	1361.7 ± 88.6c	3300.8 ± 2453.9d	178.4 ± 16.5abcd	57.3 ± 4.4bc	57.7 ± 26.4bc	14 ± 1.1cde	16.2 ± 1.2bc	22.1 ± 11.5bc	17.27 ± 2.72ab	4.29 ± 0.63ab	4.37 ± 0.6a	–0.49 ± 0.04a	–0.246 ± 0.016a	–0.46 ± 0.04ab
	LS/LV-B	2500.6 ± 183.1 cd	1273 ± 83.1c	3018.4 ± 2224 cd	175.9 ± 14.6abcd	55.5 ± 4bc	56.5 ± 24.5abc	10.2 ± 0.8ab	13.4 ± 1ab	22.4 ± 13.3bc	21.6 ± 2.72b	5.15 ± 0.63b	3.49 ± 0.6a	–0.5 ± 0.04a	–0.236 ± 0.016a	–0.42 ± 0.04abc
	HS/HV-B	2338.2 ± 188.6bcd	1307.8 ± 100.2c	2967.6 ± 2299.7bcd	153.8 ± 15.4 cd	56.8 ± 5.2bc	53.4 ± 28.2ab	13.1 ± 1bcd	15.9 ± 1.1bc	25.7 ± 10.9c	17.35 ± 2.72ab	5.21 ± 0.63b	2.88 ± 0.6a	–0.44 ± 0.04ab	–0.241 ± 0.016a	–0.42 ± 0.04abc
	HS/MV-SC	1507.1 ± 111.6a	886.3 ± 52.1ab	2371.5 ± 2115.6abc	120.6 ± 13bcd	47.3 ± 3ab	53.8 ± 27.1ab	10.6 ± 0.8abc	11.7 ± 0.8a	15.4 ± 8a	14.63 ± 2.72ab	5.3 ± 0.63b	3.93 ± 0.6a	–0.29 ± 0.04c	–0.168 ± 0.016bc	–0.36 ± 0.04bcd
	LS/LV-SC	1377.2 ± 108.8a	804.5 ± 47a	2014.2 ± 2023.9a	112.2 ± 11.4d	45.4 ± 2.8ab	49.1 ± 24.3ab	8.8 ± 0.7a	10.9 ± 0.8a	15.6 ± 16.3a	15.55 ± 2.72ab	5.32 ± 0.63b	4.74 ± 0.6a	–0.27 ± 0.04c	–0.15 ± 0.016bc	–0.3 ± 0.04d
	HS/HV-SC	1329.2 ± 155.1a	771.5 ± 64.9a	2182.5 ± 2157.1ab	100.8 ± 14.7 cd	39.1 ± 3.4a	46.8 ± 29.5a	9.4 ± 0.8a	11.4 ± 0.9a	17.2 ± 10.6ab	13.98 ± 2.72ab	4.62 ± 0.63ab	4.15 ± 0.61a	–0.25 ± 0.04c	–0.144 ± 0.016c	–0.32 ± 0.04 cd

a The data describes the impact of carrier addition and formulation on aroma release and perception. Lowercase letters within each column indicate statistically significant differences between means across samples, as discerned by Tukey’s HSD test for pairwise comparisons. The linear mixed models considered jam formulation (HS/MV: high sugar/medium viscosity; HS/HV: high sugar/high viscosity; and LS/LV: low sugar/low viscosity), carrier (B: bread and SC: sponge cake), and their interactions as fixed effects, with participants as a random effect.

The interaction effect between carrier and jam reformulation was not significant for AUC, *I*_max_, and *T*_max_ for limonene and citral release and citrus aroma perception (Table S2). Similarly, the interaction effect between carrier and jam reformulation was not significant in the first model for composite foods (Supporting Information Table S1).

The addition of carriers (bread and sponge cake) to jams differing in formulation significantly influenced the release of limonene (AUC: *F*_(2,352)_ = 48.35, *p* < 0.001; *I*_max_: *F*_(2,352)_ = 20.48, *p* < 0.001; *T*_max_: *F*_(2,353)_ = 51.24, *p* < 0.001; *R*_i_: *F*_(2,353)_ = 5.88, *p* < 0.01; and *R*_f_: *F*_(2,352)_ = 42.28, *p* < 0.001) and citral (AUC: *F*_(2,352)_ = 43.65, *p* < 0.001; *I*_max_: *F*_(2,352)_ = 28.82, *p* < 0.001; *T*_max_: *F*_(2,353)_ = 64.85, *p* < 0.001; *R*_i_: *F*_(2,353)_ = 11.04, *p* < 0.001; and *R*_f_: *F*_(2,352)_ = 37.72, *p* < 0.001) (Supporting Information Table S2). Addition of breads to jams increased the AUC by 49% for limonene and by 21% for citral, decreased *I*_max_ by 1% for limonene and by 16% for citral, and reduced *T*_max_ by 5 s for limonene and citral compared to jams alone. The addition of sponge cakes to jams decreased the AUC by 16% for limonene and by 24% for citral, decreased *I*_max_ by 35% for limonene and citral, and reduced *T*_max_ by 8 s for limonene and by 9 s for citral compared to jam alone (Table 3). Regarding *R*_i_ for both limonene and citral, a faster increase rate was observed in jams paired with breads and sponge cakes, while the jams evaluated alone exhibited a slower increase rate. Similarly, *R*_f_ exhibited a steeper decrease in samples with carriers.

Similarly, the citrus aroma intensity perception was significantly affected by the addition of carriers [AUC (*F*_(2,351)_ = 24.79, *p* < 0.001), *I*_max_ (*F*_(2,351)_ = 11.10, *p* < 0.001), and *T*_max_ (*F*_(2,352)_ = 25.01, *p* < 0.001)] (Supporting Information Table S2). The addition of sponge cakes to jams decreased AUC by 32%, *I*_max_ by 18%, and *T*_max_ by 7 s, whereas the addition of breads to jams reduced AUC by 4% and *I*_max_ by 9% without affecting *T*_max_ (Table 3). To summarize, the addition of carriers to jams with varying formulations significantly influenced the release of limonene and citral. Addition of carriers to jams led to a decrease in *T*_max_.

Regarding the analysis of the first model, which exclusively considered composite foods (Supporting Information Table S1), the impact of the type of carrier was assessed. According to the LMM, the main effect of the carrier indicated that the addition of bread to jams increased *in vivo* release significantly more than the addition of sponge cake to the jams. The AUC of limonene increased by 78% and the AUC of citral by 61% with bread addition compared to sponge cake addition (limonene: *F*_(1,267)_ = 92.08, *p* < 0.001; citral: *F*_(1,267)_ = 89.43, *p* < 0.001). Similarly, the *I*_max_ of limonene increased by 52% and the *I*_max_ of citral by 29% (limonene: *F*_(1,268)_ = 36.16, *p* < 0.001, citral: *F*_(1,268)_ = 25.60, *p* < 0.001) with bread addition compared to sponge cake addition. The *T*_max_ of limonene increased by 3 s and the *T*_max_ of citral by 4 s (limonene: *F*_(1,268)_ = 24.21, <0.001, citral: *F*_(1,268)_ = 41.26, *p* < 0.001) with bread addition compared to sponge cake addition. The *T*_max_ of citrus aroma perception (*F* = 27.48, *p* < 0.001) was reached 7 s later with bread addition compared to sponge cake addition (Table 2). To summarize, the type of carrier had a strong influence on the *in vivo* aroma release and perception. The addition of breads to jams increased the *in vivo* release of limonene and citral more than the addition of sponge cakes to jams did. Composite foods with breads as carriers tended to display a higher citrus aroma intensity compared to composite foods with sponge cake, except for the LS/LV formulation.

In contrast to the pronounced effects of carrier addition on *in vivo* aroma release and perception, the sugar content and viscosity of the jams had only a small impact on *in vivo* aroma release and perception. The *T*_max_ of limonene (*F*_(2,352)_ = 3.72, *p* < 0.05) and the *I*_max_ of citrus aroma perception (*F*_(2,351)_ = 3.76, *p* < 0.05) were significantly influenced by jam formulation (Supporting Information Table S2). The LS/LV jam reached *T*_max_ 2 s earlier compared with the HS/MV jam. The maximum citrus aroma intensity was significantly reduced by 9% in the jam with high viscosity (HS/HV) compared with the control jam (HS/MV) (Table 3). Likewise, the initial model, which exclusively considered composite foods (Supporting Information Table S1), demonstrated a consistent trend in the formulation effect. The only distinction was the statistical significance of the *T*_max_ of limonene (*F*_(2,268)_ = 8.59, *p* < 0.01), *T*_max_ of citral (*F*_(2,268)_ = 3.72, *p* < 0.05), and *I*_max_ of citrus aroma perception (*F*_(2,267)_ = 3.11, *p* < 0.05).

Lastly, with respect to the AUC, it was observed that for the release of limonene and citral, as well as for the perception of citrus aroma, on average, HS/MV jam exhibited the highest AUC, while HS/HV formulation exhibited the lowest AUC (Table 3). However, these differences, indicative of the main effect of reformulation, were not statistically significant (Supporting Information Table S2).

## 4 Discussion

It was first hypothesized that aroma release and perception are affected by chewing rate. In this study, we showed that faster chewing rates reduced the time to reach *I*_max_ for the *in vivo* release of limonene and citral and for the perception of citrus aroma intensity. Additionally, we observed that the rate of increase in *R*_i_ was higher for a faster chewing rate. The chewing rate did not influence the AUC and *I*_max_ for the *in vivo* release of citral and limonene or the citrus aroma intensity perception. This suggests that faster chewing led to faster structural breakdown of the composite foods during mastication, leading to an earlier aroma release and perception without causing perceivable changes in aroma intensity. This emphasizes that chewing rate primarily affected the temporality and rate of the release of aroma compounds from the food matrix into the nasal cavity. This aligns with the findings of van Eck and colleagues (2021), who found that the introduction of chewing significantly impacted *T*_max_. Specifically, the carrier–mayonnaise combinations exhibited a faster *T*_max_ compared to mayonnaises consumed alone.^16^ In our study, the changes in aroma release of the jam with bread or sponge cake combinations were too subtle to produce clear changes in aroma intensity perception, which is consistent with earlier findings where differences in eating speed led to only small differences in dynamic sensory perception.^11,18,25^

Secondly, it was hypothesized that adding solid carriers to strawberry jams leads to an increase in aroma release and a decrease in aroma perception. When jams were combined with bread, the aroma release increased. This may be partly attributed to the difference in oral processing time between jams alone (15 s) and composite foods (25 s). Jams consumed alone did not require chewing and were just swirled around in the mouth not following a prescribed mastication protocol, while jam–carrier combinations required chewing to break the food down, inducing more aroma release. Our results are in line with those of Hansson et al. (2003), who observed that aroma concentrations in the nose were approximately twice as high during the chewing of pectin-containing systems compared to when these foods were held in the mouth without chewing. This phenomenon was attributed to the retention of volatiles within the food matrix and their release from the matrix depending on mastication.^26^ These results highlight the key role of carrier addition in modulating the aroma release of composite foods through the changes in the physical structure of the food matrix induced by oral processing behaviors associated with different textures. We suggest that the oral processing induced structural breakdown and increased the surface area of jam–carrier combinations, allowing a higher transfer of aroma compounds from the jam into the vapor phase compared to jams consumed alone.^16,27^

Additionally, previous works have consistently demonstrated that an increase in viscosity (semisolid systems) or hardness (gel systems) can reduce perceived aroma intensity through physicochemical mechanisms where texturing agents directly interact with VOCs, affecting their release from the food matrix.^28−30^ In our study, there was a limited effect of increased viscosity on aroma release Despite the absence of changes in aroma release, the increase in consistency was sufficient to induce a perceptible decrease in aroma intensity. This texture–flavor interaction can be explained through cognitive mechanisms.^31−33^ Kora et al. (2004) highlighted that the addition of thickening agents resulted in a diminished perception of green apple aroma, despite instrumental measurements showing no effect of viscosity on the release of hexanal, the key odorant responsible for the green apple aroma.^34^ Similarly, in a separate study, Bult et al. (2007) showed that when a creamy aroma was delivered ortho- or retro-nasally while a texture stimulus was presented in the mouth, an increase in milk viscosity led to a decrease in perceived flavor intensity.^35^

Furthermore, although *in vivo* aroma release increased upon the addition of carriers to jams, citrus aroma intensity decreased. A similar phenomenon was observed in a prior study.^16^ In this study, a positive correlation was found between aroma release and intensity perception when mayonnaises were consumed alone. However, when mayonnaises were paired with bread or potatoes, there was an enhanced release of limonene and citral into the nasal cavity during consumption, accompanied by a reduction in the perceived aroma intensity of the condiments.^16^ Similarly, our recent research illustrated that the addition of carriers such as bread and wafer significantly increased the aroma release of specific molecules in chocolate–hazelnut spread, while simultaneously diminishing their sensory perception.^36^ These consistent findings across several studies highlight the role of cognitive mechanisms induced by the integration of texture and aroma perceptions, leading to perceptual cross-modal texture–aroma interactions. It could be that cognitive effects play a role in the modulation of jam–carrier aroma perception. During food consumption, consumer perception is shaped by the way attention is distributed among sensory sensations: participants may have paid more attention to texture or chewing in the presence of carriers. In other words, when consuming composite foods, the selective focus on aroma may have been impaired by the multimodal integration of the contrasting texture brought by the carriers. These findings agree with previous studies where the addition of solid food components decreased the flavor intensity of sauces or toppings. Meinert et al. (2011) showed that the addition of gravy to vegetables (broccoli, cauliflower, and potato) reduced the flavor intensity of the vegetables.^37^ Similarly, Paulsen et al. (2012) showed that addition of sauces to salmon reduced salmon flavor intensity.^38^ Van Eck et al. (2019) showed that toppings (cheese, cream cheese, and mayonnaise) affected the sensory perception of carriers (bread and cracker).^16,39^

We acknowledge that the addition of citral and limonene introduced a citrus aroma to the strawberry jams, which might have influenced taste perception (i.e., sweetness and sourness) through cross-modal aroma–taste interactions. We did not quantify taste perception of the jams using descriptive sensory methodologies but focused on citrus aroma perception using the TI methodology. We speculate that cross-modal interactions of the citrus aroma on taste may have been experienced consistently across all jams since all jams were spiked with the same citral and limonene concentration. Given the primary focus of our study on exploring the influence of oral behavior on *in vivo* aroma release and perception, we speculate that potential cross-modal interactions are likely to be uniform across samples and do not considerably alter the overall conclusions of the study.

Participants practiced the evaluation of citrus aroma intensity while being aware of the possible differences in texture caused by the addition of different carriers (bread and sponge cake) to mitigate potential sensory dumping effects, a well-known limitation of the TI methodology.^40^ The transfer of aroma compounds into the nasal cavity follows the swallow breath.^41^*In vivo* aroma release and perception are known to increase after swallowing. The *in vivo* aroma release and TI data (Figures 2 and 3) consistently reveal an increase in citral and limonene release after swallowing, accompanied by an increase in citrus aroma intensity after swallowing. The swallow breath was most pronounced for jams consumed with breads (Figure 2) and jams consumed alone (Figure 3). This suggests that participants clearly perceived the swallow breath as an increase in citrus aroma intensity, which shows that participants were capable of evaluating citrus aroma intensity and did not dump differences in texture perception or any potential differences in taste into the assessment of citrus aroma intensity. We therefore assume that the sensory dumping effect in our study, if there was any, was small and did not impact the overall conclusions of our study.

A prescribed chewing protocol, a fixed swallow moment, and a cohort comprising Caucasian young women were used in our study to minimize interindividual differences and to maximize the effect of oral behavior and food formulation on *in vivo* aroma release and perception. Future research should consider the impact of interindividual variations such as differences in oral cavity volume, salivary flow, and composition on aroma release and perception to enhance the understanding of the interactions between individual differences and food properties on aroma release and perception. Additionally, the jam reformulation in this study was within realistic product reformulation, potentially explaining the limited impact of jam formulation on aroma release and perception observed. Nevertheless, this approach provided real-world examples of product reformulation, thereby enhancing ecological validity and showing insights into the complexity of real consumption contexts.

The findings of this study revealed that the chewing rate influenced the temporality of *in vivo* aroma release and the perception of composite foods without affecting aroma intensity perception. The addition of carriers (bread and sponge cake) to strawberry jams had different effects on aroma release and perception. Although both carriers enhanced *in vivo* aroma release, addition of breads to jams prolonged and intensified the aroma release more than addition of sponge cake to jams during mastication. This pronounced effect of carrier addition highlights the importance of investigating toppings/jams accompanied by carriers rather than in isolation as the latter approach could give an inaccurate sensory profile and misguide product development.

This study stressed the complexity of aroma release and sensory perception in the consumption of complex food matrices, emphasizing the multidimensional nature of these phenomena. Simultaneous exploration of aroma release and perception provided a more comprehensive understanding of the mechanisms governing aroma release and sensory perception during food consumption.

## Supporting Information Available

Effect of carrier addition, reformulation, chewing rate, and their interactions from the first linear mixed model (LMM) on area under the curve (AUC), maximum intensity (*I*_max_), time to reach maximum intensity (*T*_max_), rising slope (*R*_i_) defined as rate of intensity increase (linear fit of data from 0 s to *T*_max_), and declining slope (*R*_f_) defined as rate of intensity decrease (linear fit of data from *T*_max_ to baseline), obtained from release of limonene, citral, and citrus aroma perception; effect of carrier addition, reformulation, and their interaction from the second linear mixed model (LMM) on area under the curve (AUC), maximum intensity (*I*_max_), and time to reach maximum intensity (*T*_max_), slope (*R*_i_) defined as rate of intensity increase (linear fit of data from 0 s to *T*_max_), and declining slope (*R*_f_) defined as rate of intensity decrease (linear fit of data from *T*_max_ to baseline), obtained from release of limonene, citral, and citrus aroma perception (PDF)
