# Supplementary material for: Influence of Chewing Rate and Food Composition on in Vivo Aroma Release and Perception of Composite Foods
Source: J Agric Food Chem. 2024 Mar 13;72(12):6723–34. doi: 10.1021/acs.jafc.3c09346 (PMC10979429; doi:10.1021/acs.jafc.3c09346)
Supplement: Supplementary file 1 — jf3c09346_si_001.pdf [file jf3c09346_si_001.pdf]

Influence of chewing rate and food composition on in vivo aroma release and perception of composite foods

Karina Gonzalez-Estanol [1,2,3], Michele Pedrotti [1], Mònica Fontova-Cerdà [2], Iuliia Khomenko[1], Franco Biasioli[1], Markus Stieger [2,4]*

[1] Research and Innovation Centre, Edmund Mach Foundation; 38098 San Michele All'Adige (TN), Italy Italy
[2] Food Quality and Design, Wageningen University, 6708 WG Wageningen, The Netherlands
[3] Department of Agri-food and Environmental Sciences, I-38123 Trento University, Trento, Italy.
[4] Division of Human Nutrition and Health, 6708 WE Wageningen University, Wageningen, The Netherlands

* karina.gonzalez.estanol@gmail.com

Supplementary Materials

Table S1. Effect of carrier addition, reformulation, chewing rate and their interactions from the first linear mixed model (LMM) on area under the curve (AUC), maximum intensity (I_{max}), time to reach maximum intensity (T_{max}), rising slope (R_i) defined as rate of intensity increase (linear fit of data from 0 s to T_{max}) and declining slope (R_f) defined as rate of intensity decrease (linear fit of data from T_{max} to baseline), obtained from release of limonene, citral and citrus aroma perception.

AUC

	Limonene				Citral				Citrus aroma perception			
	NumDF	DenDF	F value	Pr(>F)	NumDF	DenDF	F value	Pr(>F)	NumDF	DenDF	F value	Pr(>F)
Carrier	1	267	92,08	0,00	1	267	89,43	<0.001	1	267	42,48	<0.001
Formula	2	267	1,70	0,19	2	267	1,18	0,31	2	267	2,14	0,12
Chewing_rate	1	276	0,28	0,60	1	274	3,45	0,06	1	269	0,03	0,86
Carrier:Chewing_rate	1	267	0,21	0,65	1	267	0,35	0,55	1	267	2,30	0,13
Formula:Chewing_rate	2	267	0,64	0,53	2	267	0,08	0,93	2	267	1,34	0,26
Carrier:Formula	2	267	0,17	0,84	2	267	0,18	0,84	2	267	0,13	0,88
arrier:Formula:Chewing_rate	2	267	0,61	0,55	2	267	0,41	0,67	2	267	3,23	<0.05

Imax

	Limonene				Citral				Citrus aroma perception			
	NumDF	DenDF	F value	Pr(>F)	NumDF	DenDF	F value	Pr(>F)	NumDF	DenDF	F value	Pr(>F)
Carrier	1	268	36,16	<0.001	1	268	25,60	<0.001	1	267	10,26	<0.01
Formula	2	268	1,96	0,14	2	268	0,96	0,38	2	267	3,11	<0.05
Chewing_rate	1	275	1,41	0,24	1	274	0,30	0,59	1	270	0,86	0,36
Carrier:Chewing_rate	1	268	0,42	0,52	1	268	0,75	0,39	1	267	0,12	0,72
Formula:Chewing_rate	2	268	1,11	0,33	2	268	0,32	0,73	2	267	0,31	0,73
Carrier:Formula	2	268	0,11	0,90	2	268	1,04	0,35	2	267	0,24	0,79
arrier:Formula:Chewing_rate	2	268	0,27	0,76	2	268	0,20	0,82	2	267	6,22	<0.01

Tmax

	Limonene				Citral				Citrus aroma perception			
	NumDF	DenDF	F value	Pr(>F)	NumDF	DenDF	F value	Pr(>F)	NumDF	DenDF	F value	Pr(>F)
Carrier	1	268	24,21	<0.001	1	268	41,26	<0.001	1	268	38,46	<0.001
Formula	2	268	8,59	<0.001	2	268	3,72	<0.05	2	268	2,10	0,12
Chewing_rate	1	275	41,96	<0.001	1	273	43,45	<0.001	1	276	10,08	<0.01
Carrier:Chewing_rate	1	268	4,90	<0.05	1	268	8,32	<0.001	1	268	0,07	0,79
Formula:Chewing_rate	2	268	0,01	0,99	2	268	0,32	0,73	2	268	1,55	0,21
Carrier:Formula	2	268	1,56	0,21	2	268	1,16	0,31	2	268	0,28	0,76
arrier:Formula:Chewing_rate	2	268	2,22	0,11	2	268	2,43	0,09	2	268	0,00	1,00

Increase rate

	Limonene				Citral				Citrus aroma perception			
	NumDF	DenDF	F value	Pr(>F)	NumDF	DenDF	F value	Pr(>F)	NumDF	DenDF	F value	Pr(>F)
Carrier	1	269	6,60	<0.05	1	268	0,22	0,64	1	268	3,34	0,07
Formula	2	269	1,22	0,30	2	268	0,54	0,58	2	268	1,13	0,33
Chewing_rate	1	275	15,51	<0.01	1	273	18,74	<0.001	1	274	8,24	<0.001
Carrier:Chewing_rate	1	269	5,96	<0.05	1	268	1,65	0,20	1	268	0,12	0,73
Formula:Chewing_rate	2	269	0,49	0,61	2	268	0,09	0,91	2	268	0,42	0,66
Carrier:Formula	2	269	0,35	0,70	2	268	1,81	0,17	2	268	2,26	0,11
arrier:Formula:Chewing_rate	2	269	0,50	0,61	2	268	2,16	0,12	2	268	0,31	0,74

Decrease rate

	Limonene				Citral				Citrus aroma perception			
	NumDF	DenDF	F value	Pr(>F)	NumDF	DenDF	F value	Pr(>F)	NumDF	DenDF	F value	Pr(>F)
Carrier	1	268	82,75	<0.001	1	267	75,43	<0.001	1	268	32,25	<0.001
Formula	2	268	1,35	0,26	2	267	0,84	0,43	2	268	2,46	0,09
Chewing_rate	1	275	0,45	0,50	1	274	1,17	0,28	1	272	0,12	0,73
Carrier:Chewing_rate	1	2	1,64	0,20	1	27	1,31	0,25	1	268	0,01	0,93
Formula:Chewing_rate	2	268	0,67	0,51	2	267	0,03	0,97	2	268	0,94	0,39
Carrier:Formula	2	268	0,35	0,71	2	267	0,34	0,71	2	268	0,05	0,95
arrier:Formula:Chewing_rate	2	268	0,99	0,37	2	267	0,60	0,55	2	268	3,21	<0.05

Table S2. Effect of carrier addition, reformulation and their interaction from the second linear mixed model (LMM) on area under the curve (AUC), maximum intensity (I_{max}) and time to reach maximum intensity (T_{max}), slope (R_i) defined as rate of intensity increase (linear fit of data from 0 s to T_{max}) and declining slope (R_f) defined as rate of intensity decrease (linear fit of data from T_{max} to baseline), obtained from release of limonene, citral and citrus aroma perception.

AUC

	Limonene				Citral				Citrus aroma perception			
	NumDF	DenDF	F value	Pr(>F)	NumDF	DenDF	F value	Pr(>F)	NumDF	DenDF	F value	Pr(>F)
Carrier	2	352	48,35	<0.00	2	352	43,65	<0.001	2	351	24,79	<0.001
Formula	2	351	0,56	0,57	2	351	0,23	0,79	2	351	1,16	0,31
Carrier: Formula	4	351	0,40	0,81	4	351	0,55	0,70	4	351	1,18	0,32

Imax

	Limonene				Citral				Citrus aroma perception			
	NumDF	DenDF	F value	Pr(>F)	NumDF	DenDF	F value	Pr(>F)	NumDF	DenDF	F value	Pr(>F)
Carrier	2	352	20,48	<0.001	2	352	28,82	<0.001	2	351	11,10	<0.001
Formula	2	352	1,56	0,21	2	352	1,03	0,36	2	351	3,76	<0.05
Carrier: Formula	4	352	0,06	0,99	4	352	0,51	0,73	4	351	1,31	0,27

Tmax

	Limonene				Citral				Citrus aroma perception			
	NumDF	DenDF	F value	Pr(>F)	NumDF	DenDF	F value	Pr(>F)	NumDF	DenDF	F value	Pr(>F)
Carrier	2	353	51,24	<0.001	2	353	64,85	<0.001	2	352	25,01	<0.001
Formula	2	352	3,72	<0.05	2	352	1,70	0,18	2	351	2,13	0,12
Carrier: Formula	4	352	1,40	0,23	4	352	0,83	0,50	4	351	0,52	0,72

Increase rate

	Limonene				Citral				Citrus aroma perception			
	NumDF	DenDF	F value	Pr(>F)	NumDF	DenDF	F value	Pr(>F)	NumDF	DenDF	F value	Pr(>F)
Carrier	2	353	5,88	<0.01	2	353	11,04	<0.001	2	352	5,76	<0.01
Formula	2	352	1,45	0,24	2	352	0,52	0,59	2	351	1,34	0,26
Carrier: Formula	4	352	0,27	0,89	4	352	1,46	0,21	4	351	1,28	0,28

Decrease rate

	Limonene				Citral				Citrus aroma perception			
	NumDF	DenDF	F value	Pr(>F)	NumDF	DenDF	F value	Pr(>F)	NumDF	DenDF	F value	Pr(>F)
Carrier	2	352	42,28	<0.001	2	352	37,72	<0.001	2	352	27,63	<0.001
Formula	2	351	0,35	0,70	2	351	0,44	0,64	2	351	0,76	0,47
Carrier: Formula	4	351	0,53	0,71	4	351	0,42	0,80	4	351	1,41	0,23
